# Paired Capillary and Venous Serum Specimen Agreement for Protein Biomarker Measurements and Risk Category Outputs in a Multi-Cancer Early Detection Tool

**DOI:** 10.3390/diagnostics16152324

**Published:** 2026-07-24

**Authors:** Hsin-Yao Wang, Wan-Ying Lin, Chenfei Zhou, Jennifer Q. L. Xu, Zayd Anwar, Jia-Ruei Yu, Michael S. Lebowitz

**Affiliations:** 1 20/20 Biolabs, Inc., Gaithersburg, MD 20877, USA; swang@2020biolabs.com (H.-Y.W.); wlin225@buffalo.edu (W.-Y.L.);; 2 Department of Laboratory Medicine, Chang Gung Memorial Hospital at Linkou, Taoyuan 333423, Taiwan; 3 School of Medicine, National Tsing Hua University, Hsinchu 300044, Taiwan; 4 Department of Medicine, University at Buffalo-Catholic Health System, Buffalo, NY 14068, USA; 5 Department of Internal Medicine, Vassar Brothers Medical Center, Poughkeepsie, NY 12601, USA

**Keywords:** capillary blood, venous blood, multi-cancer early detection, OneTest, tumor biomarkers, protein biomarkers, decentralized diagnostics

## Abstract

**Background**: Capillary blood sampling may improve access to multi-cancer early detection (MCED) by enabling less invasive and more decentralized specimen collection. However, its paired-specimen measurement agreement with conventional venous blood for protein biomarker-based MCED testing requires validation. **Objective**: The objective of this study was to evaluate agreement between capillary and venous blood specimens for OneTest protein biomarkers and to assess the exact risk category agreement of the OneTest Premium algorithm outputs generated from both specimen types. **Methods**: This two-stage study compared paired venous and capillary blood specimens. Study 1 enrolled 143 Taiwanese adults undergoing clinically indicated biomarker testing to evaluate paired-specimen measurement agreement. Study 2 involved an exploratory paired-specimen evaluation in 13 asymptomatic U.S. volunteers using an upper arm capillary blood collection device. Biomarker concentrations were compared using paired statistical testing, correlation analysis, and Bland–Altman analysis. OneTest Premium risk categories and cancer type prediction outputs were compared between specimen types. **Results**: In Study 1, no statistically significant differences were observed between capillary and venous specimens for any evaluated biomarker. Descriptive scatterplots showed strong linear associations between paired capillary and venous biomarker measurements across the observed concentration ranges. Bland–Altman analysis showed mean biases close to zero for most biomarkers, with biomarker-specific limits of agreement. In Study 2, all 12 biomarkers were successfully measured in all 13 participants. OneTest Premium risk categorization was concordant in 11 of 13 participants; discordant cases involved adjacent low-risk and mild-risk categories, with no major or very major classification errors. Given the small sample size, these findings are descriptive and preliminary. **Conclusions**: Capillary blood specimens showed paired measurement agreement with venous specimens in Study 1. The exploratory findings from Study 2 suggest that capillary-derived measurements may support the generation of OneTest Premium outputs.

## 1. Introduction

Multi-cancer early detection (MCED) tests are a promising advancement in cancer care, enabling earlier diagnosis and treatment for improved patient outcomes [1]. Accessibility is crucial for MCED tests, as frequent and timely screening can save lives [2,3]. Traditional specimen collection methods, which rely on venous blood draws in medical facilities, limit access and pose challenges, especially for underserved or remote populations [4,5,6]. The clinical rationale for capillary blood collection is consistent with the broader movement toward decentralized diagnostics. Conventional venous blood collection remains the standard approach for most laboratory testing, but it requires trained phlebotomy personnel, dedicated collection sites, and additional logistical infrastructure. These requirements may limit access to screening among individuals living in rural areas, underserved communities, or regions with limited healthcare resources. Capillary blood sampling offers a less invasive and potentially more scalable alternative, particularly when paired with standardized collection devices, remote collection workflows, and central laboratory testing.

By integrating capillary blood sampling into MCED protocols, access to early cancer detection can be broadened. This method allows for specimen collection outside clinics, without requiring phlebotomy skills or equipment, enabling supervised collection in pharmacies or self-collection at home and making diagnostic tools more widely available.

Among the emerging MCED tools, OneTest for Cancer™ stands out as a protein biomarker-based approach [7,8,9,10]. While OneTest for Cancer™ has demonstrated strong performance using traditional venous blood samples, its broader impact depends on improving accessibility for diverse populations. Evaluating the feasibility and reliability of capillary blood sampling is therefore a critical step toward expanding access and democratizing early cancer detection with OneTest for Cancer™.

Broader MCED research has pursued several blood-based strategies, including multi-biomarker tests that combine circulating tumor DNA alterations with protein biomarkers, cell-free DNA methylation approaches that seek both cancer signal detection and tissue-of-origin prediction, and serum protein panels interpreted using machine learning algorithms [10,11,12,13]. These platforms differ substantially in their biomarker composition, measurement technologies, model development populations, and intended-use settings. Consequently, performance estimates from one MCED approach should not be assumed to be directly transferable to another. Prospective studies in intended-use populations, such as PATHFINDER, have further highlighted the importance of evaluating real-world implementation in addition to diagnostic performance [14]. Within this broader context, the present study addresses a distinct pre-examination question: whether paired capillary and venous specimens provide sufficiently similar biomarker measurement results to support a protein-based MCED tool.

The present study addresses an important gap by systematically evaluating the agreement of OneTest protein biomarkers in capillary versus venous blood specimens. Additionally, it investigates the clinical utility of capillary blood sampling within the framework of OneTest for Cancer™. By clarifying the feasibility and advantages of capillary blood collection for MCED testing, this research seeks to advance efforts aimed at improving the accessibility and efficacy of early cancer detection programs.

## 2. Materials and Methods

### 2.1. Study Populations

This study was conducted in two sequential stages. In the initial phase (Study 1), we systematically evaluated the paired-specimen measurement agreement of OneTest protein biomarkers between capillary and venous blood specimens in Taiwan. Taiwanese adults were prospectively enrolled in this comparative study, with pregnant women excluded from participation. Recruitment was based on clinical indications for biomarker testing to ensure representation across a broad range of ages and clinical backgrounds. Study 1 was designed as a paired-specimen measurement agreement study rather than as a clinically characterized cancer cohort or an intended-use screening study. Age and sex were recorded under the approved study protocol. Cancer-related clinical information, including cancer status, cancer type, disease stage, treatment history, and related clinical variables, was not collected because it was not required for the prespecified objective of comparing measurements obtained from paired capillary and venous specimens. Accordingly, Study 1 was not designed to assess clinical representativeness or to evaluate whether specimen type agreement differed according to cancer-related characteristics. In the subsequent phase (Study 2), a laboratory-developed test (LDT) validation study was performed in accordance with the regulations of the College of American Pathologists at the 20/20 Biolabs central laboratory (Gaithersburg, MD, USA). Volunteers were recruited to compare venous blood samples with specimens collected using an upper arm capillary blood collection device, with an evaluation of both biomarker measurements and OneTest algorithm outputs. All U.S. volunteers were asymptomatic and represented the intended-use population for OneTest.

### 2.2. Venous and Capillary Blood Specimen Collection

Venous and capillary blood samples were concurrently collected from each subject during a single study session. Venous specimens were drawn by venipuncture following a standardized protocol with a 22-gauge needle, typically accessing the cephalic or antecubital veins, and subsequently transferred into serum separation tubes for biochemical evaluation.

During Study 1, capillary blood was obtained from the middle finger utilizing the Haiim HC-002 blade lancet device (Winnoz Technology, New Taipei City, Taiwan). To maintain procedural uniformity, lancet specifications (1.5 mm width × 2.0 mm depth) were standardized for all participants. Subsequent to puncture, capillary blood was aspirated into serum separation microtubes (Winnoz) via negative pressure generated by the Haiim HC-002 device. This approach facilitated controlled and efficient sample collection while mitigating the risks of contamination and hemolysis. A standardized protocol was employed to optimize participant safety and comfort during capillary blood collection; the participant’s hand was positioned on an accessory pillow to stabilize the puncture site and minimize movement, thereby enhancing collection efficiency.

In Study 2, capillary blood was collected from participants using the RedDrop capillary blood collection device (RedDrop Dx, Fort Collins, CO, USA). Following upper arm skin puncture with a sterile microneedle device, blood was collected into serum microtainer tubes designed for small-volume sampling. The samples were allowed to clot, centrifuged, and processed to obtain serum for measurement.

To ensure consistency and minimize pre-examination variability, standardized collection and handling procedures were followed for all participants, including controlled specimen handling, serum separation, and aliquoting prior to examination. These procedures were implemented to maintain specimen integrity and ensure reliable biomarker measurements during the validation of capillary blood as an alternative matrix to venous serum for the OneTest platform.

### 2.3. Laboratory Measurement

The collected blood samples were subjected to examination for a panel of tumor markers, including Carcinoembryonic Antigen (CEA), Cancer Antigen 19-9 (CA19-9), Cytokeratin 19 Fragment (CYFRA21-1), Alpha-fetoprotein (AFP), Total Prostate-specific Antigen (tPSA), Cancer Antigen 15-3 (CA15-3), Cancer Antigen 125 (CA125) and Human Epididymis Protein 4 (HE4). All tumor antigens including AFP, CA-125, CA15-3, CA19-9, CEA, CYFRA 21-1, HE4, and tPSA were measured on the Roche Cobas e411 using manufacturer-supplied test kits (Roche Diagnostics Corp., Indianapolis, IN, USA). Apolipoprotein A-1 (ApoA1), β2-microglobulin, C-Reactive Protein (CRP), and prealbumin (transthyretin) were measured on the Abbott Alinity C using manufacturer-supplied test kits (Abbott Diagnostics, Abbott Park, IL, USA). All tests were run in a CAP- and CLIA-certified laboratory. To ensure the reliability and accuracy of the measurements, rigorous quality control measures were implemented. Daily quality control checks were performed according to established laboratory standards, incorporating low- and high-level controls to validate assay performance and consistency.

### 2.4. OneTest Premium Algorithm

All biomarker results were processed and used as inputs for the OneTest Premium algorithm to generate cancer risk scores. For the OneTest Premium workflow, biomarker measurement results were automatically transmitted into Laboratory Information System LabDAQ (version 4.11.0.7954, CompuGroup Medical US, Owings Mills, MD, USA). From LabDAQ, a standardized DataMiner export file was created, compiling all required unique patient identifiers and the corresponding biomarker values needed for algorithm input. A transformer program was then used to convert the DataMiner file into a format compatible with the Premium algorithm’s input requirements. This formatted file was subsequently uploaded into the OneTest Premium algorithm platform, which processed the dataset to generate risk scores across multiple cancer types, including lung, liver, colorectal, stomach, breast, pancreatic, ovarian, and prostate cancers.

The quantitative biomarker inputs for the OneTest Premium workflow were measured using manufacturer-supplied in vitro diagnostic assays on the Roche Cobas e411 and Abbott Alinity C platforms. Calibration, internal quality control, and ongoing examination performance monitoring were performed according to the respective manufacturer instructions and the laboratory quality system. OneTest Premium is a proprietary interpretive algorithm that processes the biomarker measurement results to generate risk category and cancer type outputs. It is a laboratory-developed interpretive workflow rather than a validated in vitro diagnostic. Detailed model parameters, coefficients, decision thresholds, and software implementation are proprietary and maintained within the company’s controlled quality and regulatory documentation. These details are therefore not fully disclosed in the present article. 20/20 Biolabs, Inc., a Nasdaq-listed company (Nasdaq: AIDX), is commercializing these algorithms under the OneTest® brand.

The present paired-specimen study evaluated the effect of specimen source on biomarker measurement results and on the generation of algorithm outputs. It was not intended to provide a complete public analytical validation report of the proprietary algorithm. The same locked algorithm version and processing workflow were applied to each paired venous and capillary specimen set.

### 2.5. Institutional Approval

Ethical approval for the study protocol was obtained from the Institutional Review Board (IRB) of Chang Gung Memorial Hospital (IRB No. 202301302B0), ensuring compliance with ethical guidelines and safeguarding the welfare of study participants. All participants provided informed consent prior to enrollment, affirming their voluntary participation in this study.

### 2.6. Statistical Analysis

Statistical analysis was conducted to compare the results obtained from venous and capillary blood samples for each biomarker. Descriptive statistics, including means, standard deviations, and medians, were calculated to summarize the data. Additionally, correlation coefficients and Bland–Altman analysis were employed to assess the agreement between venous and capillary blood samples [15]. Data analysis was performed using appropriate statistical software, with significance set at a *p* value < 0.05.

Descriptive summaries, paired statistical comparisons, and scatterplots with identity and ordinary least-squares regression lines were used to visualize the distribution and relationship of paired capillary and venous measurement results. These analyses were considered descriptive and were not used to establish paired measurement agreement. A primary assessment of paired measurement agreement was performed using Bland–Altman analysis, including the estimation of mean paired bias and the 95% limits of agreement for each biomarker.

## 3. Results

### 3.1. Study 1

The study cohort consisted of 143 adults with diverse demographic characteristics and clinical backgrounds. Participants were recruited from multiple clinical departments and underwent blood collection through the centralized blood draw center. Recruitment based on clinical indications for tumor marker testing enabled the inclusion of individuals across a broad spectrum of ages and medical conditions, thereby generating a heterogeneous population reflective of real-world clinical practice. The cohort included individuals with varying clinical histories and disease states, allowing for the assessment of paired-specimen measurement agreement under diverse physiological and pathological conditions. This broad representation enhances the generalizability of the findings and supports the evaluation of capillary blood sampling performance across a clinically relevant population.

### 3.2. Result Comparison of Biomarkers

**Figure 1:**
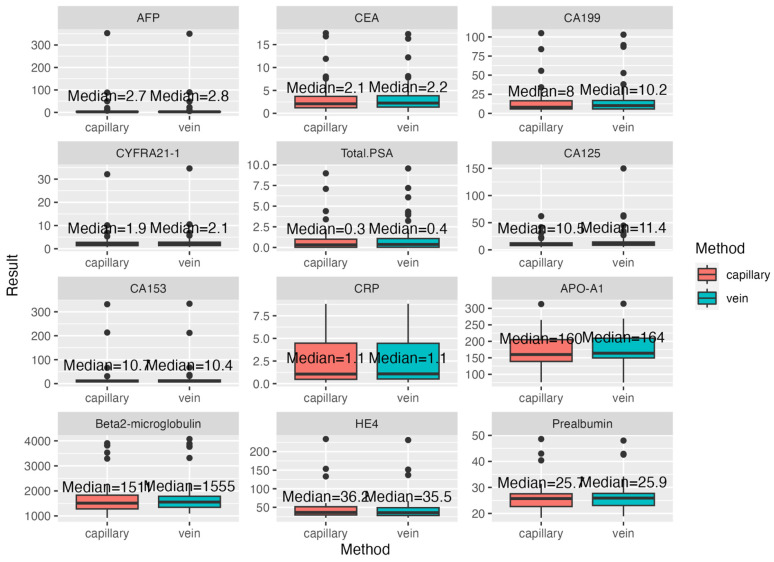
Comparison of biomarker concentrations between capillary and venous blood samples. Boxplots show distribution of measured biomarker levels in paired capillary and venous blood specimens across 12 biomarkers, including AFP, CEA, CA19-9, CYFRA21-1, total PSA, CA125, CA15-3, CRP, ApoA1, β2-microglobulin, HE4, and prealbumin. Red boxes represent capillary blood measurements, and blue boxes represent venous blood measurements. Horizontal line inside each box indicates median, with median values annotated within each panel. Overall, median concentrations were visually comparable between capillary and venous samples across most biomarkers, supporting feasibility of capillary blood as alternative specimen type for multi-biomarker testing.

Comparative analyses were conducted to assess the agreement of biomarker measurements obtained from venous and capillary blood specimens. Across all evaluated biomarkers, no statistically significant differences were observed between the two specimen types (Table 1 and Figure 1). Mean biomarker concentrations were highly comparable between capillary and venous samples across a wide range of values, indicating consistency across collection methods.

### 3.3. Result Comparison

**Figure 2:**
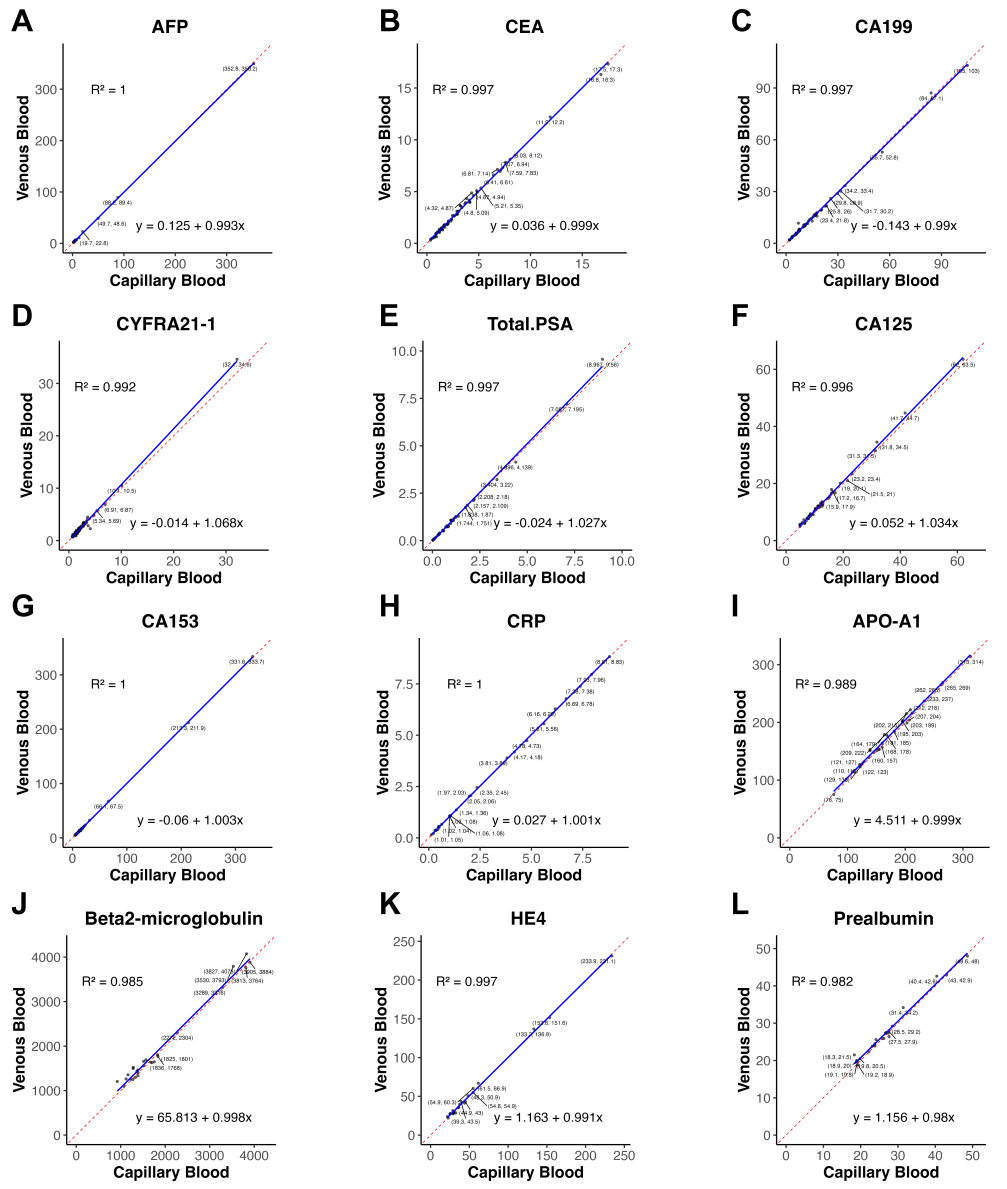
Descriptive scatterplots of paired capillary and venous biomarker measurements in Study 1. Panels show paired measurements for (**A**) AFP, (**B**) CEA, (**C**) CA19−9, (**D**) CYFRA 21−1, (**E**) total PSA, (**F**) CA125, (**G**) CA153, (**H**) CRP, (**I**) APO−A1, (**J**) beta2−microglobulin, (**K**) HE4, and (**L**) prealbumin. Each point represents one participant with paired capillary and venous specimens; axes are shown in assay-specific measurement units. The red dashed line represents the line of equality (venous measurement = capillary measurement). The blue line represents the ordinary least-squares fitted regression line. Regression equations and coefficients of determination (R^2^) are shown for the descriptive visualization of the relationship between specimen measurements only.

To further evaluate the relationship between measurements obtained from venous and capillary blood specimens, correlation analyses were performed for all biomarkers. Strong positive correlations were observed across all evaluated biomarkers, with coefficients of determination (R^2^ values) exceeding 0.99 (Figure 2). These findings indicate an exceptionally high degree of correlation between the two sampling methods.

### 3.4. Bland–Altman Analysis

**Figure 3:**
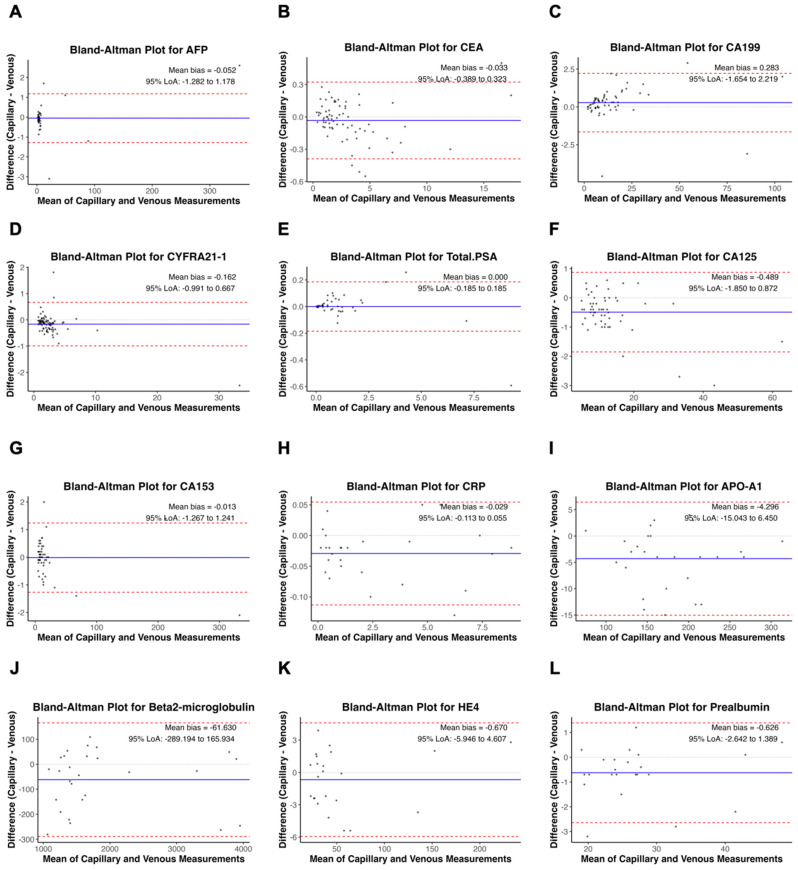
Bland–Altman plots of paired capillary and venous biomarker measurements in Study 1. Panels show the results for (**A**) AFP, (**B**) CEA, (**C**) CA19−9, (**D**) CYFRA 21−1, (**E**) total PSA, (**F**) CA125, (**G**) CA153, (**H**) CRP, (**I**) APO−A1, (**J**) beta2−microglobulin, (**K**) HE4, and (**L**) prealbumin. Each point represents one paired capillary and venous measurement. The x-axis represents the mean of the paired measurements, and the y-axis represents the paired difference calculated as capillary minus venous measurement. The solid blue line indicates the mean paired difference (bias), the red dashed lines indicate the lower and upper 95% limits of agreement (mean bias ± 1.96 standard deviations of the paired differences), and the gray dotted line indicates zero difference. Mean bias and 95% limits of agreement are displayed in each panel. Agreement between venous and capillary measurements was further examined using Bland−Altman analysis. The results demonstrated narrow limits of agreement and minimal systematic bias across all biomarkers evaluated. Most paired measurements fell within the expected agreement boundaries, indicating strong consistency and the absence of substantial directional bias between sampling methods.

Bland–Altman plots were used to describe paired differences between capillary and venous measurements for each biomarker (Figure 3). Differences were calculated as capillary minus venous measurements and plotted against the mean of the paired measurements.

Across the 12 biomarkers, mean biases were generally close to zero, indicating minimal systematic differences between capillary and venous measurements overall. However, the magnitude and dispersion of paired differences varied by biomarker and across the measurement range. For most biomarkers, the differences were tightly clustered around the mean bias with relatively narrow limits of agreement, suggesting good agreement between sampling methods. A small number of outliers were also observed for certain biomarkers, where individual paired differences fell outside the 95% limits of agreement.

Together, these findings demonstrate that capillary blood collection yields biomarker measurements closely aligned with those obtained from conventional venous sampling. The combination of minimal measurement differences, narrow agreement limits, and a lack of systematic bias further supports the analytical interchangeability of capillary and venous blood specimens for biomarker measurement and multi-cancer early detection applications.

### 3.5. Study 2

Samples from all 13 participants were successfully analyzed for the complete panel of 12 biomarkers, with no missing data across the study cohort. The result comparison demonstrated strong agreement in biomarker measurements between venous and capillary specimens, with coefficients of variation (CVs) below 6.4% for all evaluated biomarkers. These findings indicate high reproducibility and minimal result variability between specimen collection methods.

### 3.6. Exploratory Cross-Classification of OneTest Premium Risk Categories

The OneTest Premium predictive outputs generated from biomarker measurements obtained from venous and capillary blood specimens demonstrated a high degree of exact risk category agreement (Table 2). Overall risk categorizations showed agreement in 11 of 13 participants, indicating strong consistency between the two specimen types. The two discordant cases involved differences between adjacent risk categories, specifically between low-risk and mild-risk classifications. Importantly, no discordant results involved clinically more consequential classification shifts, such as major errors (low- or mild-risk samples classified as moderate-risk) or very major errors (moderate-risk samples classified as low- or mild-risk). However, because of the small size of the exploratory cohort, these findings should not be interpreted as a definitive estimate of risk category agreement or as evidence of clinical equivalence between specimen types.

### 3.7. Exploratory Within-Participant Comparison of Cancer Type Outputs

In an exploratory within-participant comparison involving one female participant, six of seven cancer type output categories were identical between venous and capillary specimens (Supplementary Table S1). This single-participant observation is descriptive only and cannot be used to estimate cancer type output agreement. The sole discrepancy involved the ovarian cancer prediction output. A detailed examination of the raw algorithm probability scores revealed that both values were positioned near the predefined decision threshold separating non-detected and detected categories. Furthermore, a review of the corresponding biomarker measurements showed no substantial variation between venous and capillary specimens (23.25 U/mL vs. 21.95 U/mL), indicating that the discordance was not driven by meaningful result differences in biomarker values. Instead, the discrepancy likely reflected sensitivity to threshold-based categorization when scores are located near classification boundaries. Such findings suggest that the observed variation was attributable to algorithm cutoff effects rather than limitations in specimen types.

Collectively, the Study 2 observations show that complete biomarker measurements and OneTest Premium outputs could be generated from paired capillary specimens in this small cohort. The observed risk category and cancer type output comparisons are preliminary and descriptive. They do not establish algorithm robustness, category-specific agreement, or clinical equivalence across specimen types. Larger prospective studies are required before capillary-derived algorithm outputs can be interpreted as reproducible or interchangeable with venous-derived outputs.

## 4. Discussion

The present study demonstrates that capillary blood specimens can provide biomarker measurements and OneTest Premium algorithm outputs that are highly concordant with those obtained from conventional venous blood specimens. In the Taiwanese cohort, no statistically significant differences were observed between venous and capillary specimens for the evaluated biomarkers, and paired measurements showed strong correlation across biomarkers. In the small U.S. exploratory cohort, all 12 biomarkers were successfully measured in all 13 participants, with low analytical variation between specimen types. OneTest Premium risk categorization was concordant in 11 of 13 participants, and the two discordant cases were limited to adjacent low-risk and mild-risk categories, without major or very major classification errors. Collectively, these observations provide initial operational evidence that algorithm outputs can be generated from capillary-derived measurements.

### 4.1. Capillary Blood Sampling as a Decentralized Specimen Collection Strategy

Recent studies support the feasibility of capillary blood sampling for laboratory-based testing, although the paired-specimen measurement agreement varies by measurand, platform, and pre-examination workflow. Parikh et al. evaluated a novel capillary blood collection system in nontraditional settings and demonstrated clinical equivalence for selected general chemistry measurements when compared with marketed capillary and venous blood collection systems [16]. Nwankwo et al. further showed that remote capillary blood testing could be incorporated into outpatient care pathways and assessed agreement with venous sampling using paired comparisons [17]. In addition, El-Sabawi et al. demonstrated that capillary blood self-collection can support high-throughput proteomic profiling, which is particularly relevant to biomarker-intensive diagnostic applications [18]. The present study extends this literature by evaluating biomarkers and downstream algorithmic cancer risk outputs rather than routine chemistry or broad proteomic measurements.

### 4.2. Comparison with the Prior Capillary Blood and Microsampling Literature

The results observed in the present study are broadly consistent with the emerging evidence that modern capillary collection systems can generate clinically meaningful laboratory data. However, prior studies also emphasize that capillary blood should not be assumed to be interchangeable with venous blood without analyte-specific validation. Dried blood spot and microsampling approaches have been widely discussed as strategies for expanding diagnostic access and simplifying sample transport, but they differ from liquid capillary blood collection in sample matrix, processing requirements, and examination constraints [19,20]. Therefore, although dried blood spot studies provide useful context regarding decentralized sampling, they should be interpreted as indirect evidence rather than direct support for liquid capillary biomarker testing.

Pre-examination variability remains a central concern. Bond and Richards-Kortum reported drop-to-drop variation in the cellular components of fingerprick blood, highlighting the importance of standardized collection procedures, adequate sample volume, and validated devices [21]. This issue is especially important for algorithm-based diagnostics, where small laboratory differences may influence categorical outputs when values are close to decision thresholds. In the present study, the only discordant cancer type prediction involved the ovarian cancer algorithm output in one female participant (Supplementary Table S1). A review of the raw probability scores indicated that both the venous and capillary results were located near the threshold separating non-detected and detected categories. The underlying biomarker values showed minimal difference, suggesting that the discordance reflected threshold sensitivity rather than meaningful analytical disagreement. This finding supports the need for the predefined handling of borderline algorithm scores in future clinical implementation.

### 4.3. Relevance to Protein-Based MCED Testing

The present findings are particularly relevant to protein-based MCED strategies. Many MCED platforms have emphasized circulating tumor DNA, methylation signatures, or multi-omic approaches. In contrast, OneTest uses established serum protein biomarkers combined with algorithmic interpretation. Protein-based approaches may offer practical advantages, including lower technical complexity, compatibility with existing immunoassay infrastructure, and potentially lower cost. Luan et al. reported that a seven-protein tumor marker panel combined with artificial intelligence improved multi-cancer detection performance compared with conventional single-marker threshold interpretation, illustrating the potential of algorithm-enhanced protein marker panels for affordable MCED applications [11]. Budnik et al. similarly described a proteomics-based plasma test for the early detection of multiple cancers, further supporting the broader concept that circulating protein signatures may contribute meaningfully to MCED development [22].

The current study adds a specimen dimension that is largely absent from much of the MCED literature. While prior protein-based MCED studies have evaluated diagnostic discrimination, cancer type prediction, and model performance, most have relied on venous blood or plasma specimens. By demonstrating that capillary-derived biomarker measurements can produce highly concordant OneTest algorithm outputs, this study suggests that protein-based MCED may be compatible with less invasive and more decentralized blood collection workflows. This could be especially important for screening programs targeting asymptomatic individuals, for whom convenience and accessibility are critical determinants of participation.

### 4.4. Comparison with the Broader MCED Literature

Several landmark MCED studies provide important context for interpreting the present findings. Cohen et al. introduced CancerSEEK, a multi-measurand blood test combining circulating tumor DNA mutations and protein biomarkers to detect eight common cancer types [12]. Lennon et al. subsequently evaluated a blood-based multi-cancer screening strategy combined with PET-CT confirmation in the DETECT-A study, demonstrating the feasibility of incorporating blood-based multi-cancer testing into a prospective screening workflow [17]. Methylation-based MCED approaches have also shown substantial progress. Liu et al. reported sensitive and specific multi-cancer detection and localization using methylation signatures in cell-free DNA [13], while Klein et al. provided independent validation of a targeted methylation-based MCED test [23]. The PATHFINDER study evaluated a blood-based MCED test prospectively in an intended-use screening population and emphasized both feasibility and the need for additional evidence regarding clinical utility [14].

Compared with these studies, the present investigation addresses a narrower but essential question: whether capillary blood can serve as an analytically comparable specimen source for a protein-based MCED algorithm. This study was not designed to estimate cancer detection sensitivity, specificity, positive predictive value, mortality impact, or downstream diagnostic burden. Therefore, the present results should be interpreted as evidence of specimen and algorithmic stability, rather than evidence of clinical effectiveness. This distinction is important because MCED implementation requires multiple layers of validation, including analytical validity, clinical validity, clinical utility, patient acceptability, and health system feasibility.

### 4.5. Considerations on CYFRA 21-1 Variability

Although overall agreement between venous and capillary specimens was strong, the observation of larger differences in CYFRA 21-1 at higher concentration ranges warrants further attention (Figure 3D). In the present dataset, these differences did not alter prediction categories. However, concentration-dependent variability may become clinically relevant in larger cohorts, particularly for measurands with nonlinear assay behavior, matrix effects, or values near algorithmic decision thresholds. Future studies should therefore include sufficient representation of samples across the full measurement range, including high-positive specimens.

### 4.6. Implications for Healthcare Delivery

Capillary blood sampling may improve the scalability and accessibility of MCED testing. By reducing reliance on conventional venipuncture and centralized phlebotomy visits, capillary collection could support home-based, community-based, or workplace-based screening workflows. This may be particularly valuable for individuals with limited access to preventive care, those living far from medical centers, and populations with low participation in conventional cancer screening. If examination performance is maintained, decentralized collection may reduce logistical barriers and improve the uptake of MCED testing.

At the same time, decentralized sampling introduces new operational challenges. Collection adequacy, sample volume, hemolysis, clotting, transport time, temperature control, patient instruction, device usability, and chain-of-custody procedures must be carefully standardized. For algorithm-based MCED testing, laboratory reports should also address borderline or near-threshold results transparently. The discordant ovarian cancer prediction observed in this study (Supplementary Table S1) illustrates that categorical outputs may change when raw scores are close to cutoffs, even when biomarker measurements are highly similar. Future reporting frameworks may need to include gray-zone interpretation, repeat-testing recommendations, or probability-based reporting rather than relying exclusively on binary category labels.

## 5. Limitations

Several limitations should be acknowledged. First, Study 1 was designed as a paired-specimen measurement agreement study, and cancer-related clinical information, including cancer status, cancer type, disease stage, and treatment history, was not collected under the approved protocol. Accordingly, we could not assess whether agreement between capillary and venous specimens differed according to these characteristics, nor can the cohort be considered representative of a clinically defined cancer cohort or an intended-use screening population. The findings should therefore be interpreted as evidence of paired measurement agreement in the enrolled adult cohort rather than as evidence of clinical performance in a cancer or screening population. Second, Study 2 included only 13 volunteers and was exploratory. Consequently, the observed exact risk category agreement of 11 of 13 participants and the 2 of 13 discordant classifications should not be interpreted as stable estimates of agreement. Likewise, the cancer type output comparison in a single participant cannot support conclusions regarding cancer type output reproducibility or clinical relevance. Third, the number of cancer-positive cases and high-biomarker specimens may be insufficient to fully evaluate performance across the complete clinical and laboratory spectrum. Fourth, this study primarily assessed paired-specimen measurement agreement rather than clinical sensitivity, specificity, positive predictive value, or cancer outcome endpoints. Finally, capillary sampling performance may depend on the specific collection device, anatomical site, collection protocol, sample volume, transport conditions, and laboratory workflow. Therefore, the present findings should not be generalized to all capillary collection methods without additional validation.

### Future Directions

Future studies should validate capillary blood sampling for OneTest in larger prospective screening cohorts, ideally including asymptomatic intended-use populations, cancer-positive participants, and individuals with benign conditions that may affect biomarker levels. Longitudinal studies are also needed to evaluate whether capillary sampling supports the serial monitoring of biomarker patterns over time. Because MCED algorithms may be sensitive to small changes near decision thresholds, future validation should include predefined analyses of borderline results, repeatability, inter-device variability, and inter-operator variability. Studies should also evaluate the patient acceptability, real-world collection success rates, sample rejection rates, and cost-effectiveness of decentralized capillary collection compared with standard venipuncture workflows.

## 6. Conclusions

In summary, Study 1 provides paired-specimen measurement agreement data for capillary and venous biomarker measurements. Study 2 provides preliminary exploratory evidence that OneTest Premium outputs can be generated from capillary-derived biomarker measurements. The findings are consistent with emerging evidence supporting capillary blood collection for decentralized laboratory testing and extend prior work by evaluating a protein-based MCED algorithm. Although further studies are required to confirm performance in larger prospective screening populations and to define best practices for borderline algorithm scores, the present results support capillary blood sampling as a feasible and potentially scalable specimen collection strategy for MCED implementation.

## Figures and Tables

**Table 1:** Comparison of biomarker concentrations between capillary and venous blood samples in Study 1 (**A**) and Study 2 (**B**). Data are presented as mean (standard deviation, SD). Biomarker levels obtained from capillary and venous blood specimens were compared to evaluate agreement and potential differences between sampling methods. *p* values were calculated using paired statistical analysis comparing matched capillary–venous sample pairs. Paired *p* values are provided as descriptive comparisons and should not be interpreted as evidence of measurement agreement, equivalence, or interchangeability between specimen types.

Study 1 (A)	Capillary	Vein	*p*
Gender = Male (%)	76 (53.1%)		
Age	53.22 (8.64)		
Total PSA (mean (SD))	0.86 (1.58)	0.95 (1.63)	0.736
CYFRA 21-1 (mean (SD))	2.59 (3.64)	2.69 (3.79)	0.859
AFP (mean (SD))	11.04 (45.10)	9.02 (38.98)	0.77
CA125 (mean (SD))	12.94 (9.66)	15.48 (18.36)	0.349
CA153 (mean (SD))	21.63 (51.29)	18.61 (41.94)	0.705
CA199 (mean (SD))	13.83 (17.11)	15.28 (17.96)	0.62
CEA (mean (SD))	3.07 (3.19)	3.11 (3.02)	0.935
CRP (mean (SD))	2.63 (2.76)	2.66 (2.76)	0.969
APO-A1 (mean (SD))	174.52 (52.77)	178.81 (52.99)	0.767
Beta2-microglobulin (mean (SD))	1859.85 (928.33)	1921.48 (933.49)	0.809
HE4 (mean (SD))	54.44 (50.96)	52.17 (48.23)	0.873
Prealbumin (mean (SD))	26.77 (7.75)	27.40 (7.66)	0.784
Study 2 (B)	Capillary	Vein	*p*
Gender = Male (%)	7 (53.8%)		
Age	50.46 (11.61)		
Total PSA (mean (SD))	1.18 (1.54)	1.25 (1.65)	0.933
CYFRA 21-1 (mean (SD))	1.62 (0.95)	1.51 (0.61)	0.741
AFP (mean (SD))	2.97 (2.45)	3.39 (2.51)	0.668
CA125 (mean (SD))	13.24 (12.79)	13.73 (12.67)	0.922
CA153 (mean (SD))	14.46 (5.60)	14.76 (6.10)	0.920
CA199 (mean (SD))	13.91 (12.12)	14.84 (12.32)	0.848
CEA (mean (SD))	1.96 (1.51)	2.03 (1.41)	0.906
CRP (mean (SD))	0.15 (0.13)	0.15 (0.12)	0.875
APO-A1 (mean (SD))	161.38 (32.58)	165.54 (32.62)	0.748
Beta2-microglobulin (mean (SD))	1.38 (0.20)	1.44 (0.22)	0.483
HE4 (mean (SD))	46.73 (11.24)	47.79 (12.47)	0.821
Prealbumin (mean (SD))	28.62 (3.80)	29.92 (3.55)	0.373

**Table 2:** Agreement in predictive categories between venous and capillary blood samples. Values are numbers of participants. Exact category agreement was observed in 11 of 13 participants (84.6%); 2 of 13 participants (15.4%) had discordant adjacent low-risk and mild-risk categories. C\V stands for capillary specimen and venous specimen.

C\V	Low	Mild	Moderate	Total
Low	5	1	0	6
Mild	1	1	0	2
Moderate	0	0	5	5
Total	6	2	5	13

## Data Availability

The raw operational data are not publicly available due to institutional confidentiality and regulatory requirements. De-identified extracts and/or aggregated outputs supporting the findings can be made available from the corresponding author upon reasonable request.

## Supplementary Materials

The following supporting information can be downloaded at https://www.mdpi.com/article/10.3390/diagnostics16152324/s1, Table S1: Biomarker results and OneTest Premium Scores for the US volunteers.
